# Heme oxygenase-1 activation in mononuclear phagocytes in acute Kawasaki disease

**DOI:** 10.3389/fped.2026.1848785

**Published:** 2026-06-03

**Authors:** Jieqiong Zeng, Yizheng Huang, Tao Chen, Bofei Li, Ke Wei, Anna Qiao, Xianjuan Shen, Zhiyuan Tang, Jianmei Zhao

**Affiliations:** 1 Department of Pediatrics, Affiliated Hospital of Nantong University, Medical School of Nantong University, Nantong, Jiangsu Province, China; 2 Department of Clinical Laboratory, Affiliated Hospital of Nantong University, Nantong, Jiangsu Province, China; 3 Department of Pharmacy, Affiliated Hospital of Nantong University, Nantong, Jiangsu Province, China

**Keywords:** bulk RNA-seq, HMOX1, inflammation, kawasaki disease, mononuclear phagocytes, scRNA-seq

## Abstract

**Introduction:**

Kawasaki disease (KD) is an acute systemic vasculitis with an unknown etiology, and the underlying molecular mechanisms of vascular inflammation remain unclear. Mononuclear phagocyte activation is a prominent feature of the acute phase, but the upstream regulators of this process remain incompletely understood. This study aimed to investigate the role of heme oxygenase-1 in mononuclear phagocyte inflammation in KD.

**Methods:**

Integrating single-cell RNA sequencing (scRNA-Seq) and bulk RNA sequencing (bulk RNA-Seq) data, we analyzed differences in peripheral blood immune cell composition and key molecular expression profiles in children with KD to identify potential targets linked to mononuclear phagocyte activation. We collected clinical samples from KD patients to measure HMOX1 expression in peripheral blood mononuclear cells (PBMCs) and corresponding changes in serum inflammatory cytokines for validation. A KD mouse model was established via intraperitoneal injection of CAWS, and a separate therapeutic model was generated using SnMP. We then employed western blot, hematoxylin and eosin (HE) staining, cardiac ultrasound, and immunohistochemical staining to assess HMOX1 expression, coronary artery inflammatory infiltration, changes in coronary artery internal diameter, and macrophage infiltration.

**Results:**

Integrated analysis of single-cell and bulk RNA sequencing data showed elevated HMOX1 expression in mononuclear phagocytes from KD patients, correlating with reactive oxygen species and nuclear factor kappa B signaling pathways. In clinical samples, HMOX1 expression was increased in peripheral blood mononuclear cells, and serum tumor necrosis factor-alpha levels were elevated. In KD murine models, perivascular inflammatory infiltration, mononuclear phagocyte infiltration, and increased expression of HMOX1 in mononuclear phagocytes were observed, accompanied by elevated tumor necrosis factor-alpha expression. Notably, treatment with SnMP attenuated these pathological changes.

**Conclusions:**

Our study suggests that HMOX1 is upregulated in mononuclear phagocytes during acute Kawasaki disease and is associated with activation of inflammatory pathways. The HMOX1 inhibitor SnMP alleviated vascular inflammatory injury, indicating its potential as a therapeutic target for KD.

## 1 Introduction

Kawasaki disease (KD) is an acute systemic vasculitis that predominantly affects children under five years of age, targeting medium and small arteries, particularly the coronary arteries (1, 2). Without timely treatment, approximately 15%–25% of patients may develop coronary artery lesions, which can result in long-term cardiovascular sequelae (3). Despite the efficacy of high-dose intravenous immunoglobulin (IVIG) in conjunction with aspirin in enhancing prognosis, a portion of patients exhibits resistance to IVIG, therefore remain at risk of recurrence or delayed cardiovascular problems. Patients resistant to IVIG exhibit a markedly increased risk of developing coronary artery aneurysms (4, 5).

Although the pathogenesis of KD remains unclear, growing evidence suggests that mononuclear phagocytes play a central role in inflammatory amplification and vascular injury (6, 7). These cells can infiltrate the vascular wall, release proinflammatory mediators, and contribute to tissue remodeling and injury (8–12). The aberrant production of pro-inflammatory cytokines such as TNF-α, IL-1, and IL-6 drives many inflammatory autoimmune conditions, such as KD and systemic lupus erythematosus (13). However, the molecular programs that sustain their activated state remain incompletely understood.

HMOX1 is the rate-limiting enzyme in heme catabolism, catalyzing heme degradation into ferrous iron, CO, and biliverdin; biliverdin is subsequently converted to bilirubin (14–16). Although it is traditionally regarded as a stress-responsive cytoprotective molecule, its function appears to be highly context-dependent (17–19). Under inflammatory and oxidative stress conditions, increased HMOX1 activity may promote reactive oxygen species generation and inflammatory signaling (20–24). Whether HMOX1 contributes to the proinflammatory state of mononuclear phagocytes in KD remains unclear.

To investigate the role of HMOX1 in mononuclear phagocyte activation in KD, we integrated single-cell transcriptomic analysis, independent whole-blood transcriptomic cohorts, clinical sample validation, and a CAWS-induced murine vasculitis model. This study aimed to determine whether HMOX1 is enriched in mononuclear phagocytes, whether it is associated with inflammatory signaling, and whether pharmacologic inhibition of heme oxygenase attenuates vascular inflammation *in vivo*.

## 2 Materials and methods

### 2.1 Data acquisition

In this study, the KD-related datasets, including GSE168732, GSE68004, and GSE73461, were downloaded from the NCBI Gene Expression Omnibus (GEO; https://www.ncbi.nlm.nih.gov/geo/) database. Data from the GSE168732 dataset, comprising single-cell RNA sequencing (scRNA-seq), were acquired, including 6 samples collected before IVIG treatment, 6 after IVIG treatment, and 3 from healthy controls. Bulk transcriptomic data from the GSE73461 dataset, comprising 77 KD samples and 55 healthy controls, were also obtained. Furthermore, we obtained transcriptomic data from the GSE68004 dataset, which includes 76 KD samples and 37 healthy controls.

### 2.2 Single-cell data quality control

Expression matrices were imported into R using the Seurat program. Cells were filtered according to three criteria: the quantity of unique molecular identifiers per cell, the number of genes identified per cell, and the proportion of mitochondrial gene expression. Elevated mitochondrial content, combined with diminished RNA expression, frequently indicates apoptotic or low-quality cells. We implemented quality control using the median absolute deviation (MAD) and excluded cells whose measures diverged by more than 3 MADs from the median. Potential doublets were subsequently detected and removed from each sample using the DoubletFinder program (version 2.0.4) (33), thereby finalizing the initial quality control.

### 2.3 Single-cell data dimensionality reduction, clustering, and annotation

The total expression of each cell was scaled to 10,000 using the LogNormalize method with a scale factor of 10,000, followed by log transformation. Cell cycle scores were calculated using the CellCycleScoring function. We identified highly variable genes using the FindVariableFeatures function. We applied the ScaleData function to regress out sources of variation from mitochondrial and ribosomal gene expression percentages and cell cycle phase differences. Principal component analysis (PCA) was performed on the expression matrix using the RunPCA function for linear dimensionality reduction, and selected principal components were used for downstream analyses. Batch effects were removed with Harmony. Nonlinear dimensionality reduction and two-dimensional visualization of global cell relationships were achieved using the RunUMAP function in the Uniform Manifold Approximation and Projection (UMAP) package. The cell annotation process involved identifying tissue-specific cell types and their marker genes, primarily by querying the Cell Marker and PanglaoDB databases and relevant literature (25, 26), with supplementary automated annotation from the SingleR software.

### 2.4 Cell-cell communication analysis

CellChat quantitatively infers intercellular communication networks from single-cell data (27). This tool uses network analysis and pattern recognition to infer significant signaling inputs and outputs in cells, and to assess the coordination of cellular processes and signals. The study used normalized single-cell expression profiles, with cell subtypes determined from the single-cell analysis, which provided the cellular information. CellChat was employed to examine interactions across various cell subtypes, evaluating the proximity of these links via interaction strength (weight) and interaction frequency (count) to evaluate the activity level and influence of each cell type within the disease context.

### 2.5 Batch effect correction for integrated datasets

We combined two KD transcriptomic datasets (GSE73461 and GSE68004) from the GEO database, comprising samples from the control and disease groups, respectively. The ComBat algorithm, which employs an empirical Bayes technique, was used to correct batch effects from the disparate datasets in the merged expression matrix (28). Principal component analysis (PCA) was used to visualize the samples pre- and post-correction to assess the efficacy of batch-effect elimination.

### 2.6 Co-expression analysis

The co-expression profile of HMOX1 in KD data was examined using correlation coefficients and *p*-values of 0.2 and 0.05, respectively. The ten genes most strongly associated with HMOX1 expression were discovered, and their correlations were illustrated using the “corrplot” and “circlize” packages to create correlation circle plots and heatmaps.

### 2.7 Immune cell infiltration analysis

The immune-cell composition in the microenvironment was assessed using the CIBERSORT technique. This method employs a support vector regression-based deconvolution algorithm on expression matrices, utilizing a signature matrix of 547 biomarkers to measure the relative proportions of 22 human immune cell phenotypes, such as T cells, B cells, plasma cells, and myeloid subsets (29). We used the CIBERSORT algorithm on RNA-seq data from patients with KD to estimate the proportions of 22 immune cell types. Subsequent correlation studies evaluated the association between gene expression levels and immune cell concentrations, with *p*-values < 0.05 considered statistically significant.

### 2.8 Gene set enrichment analysis (GSEA)

Patients were categorized into high- and low-expression groups based on the expression levels of the key gene. GSEA was subsequently conducted to discern pathway discrepancies between these groups, utilizing the version 7.0 annotated gene set from the MsigDB database as the reference for pathway annotation. Pathways were prioritized based on enrichment scores from differential expression analysis, with significantly enriched gene sets defined by an adjusted *p*-value < 0.05. This approach is often used to clarify the biological importance linked to disease subtypes.

### 2.9 Gene set variation analysis (GSVA)

GSVA is a nonparametric, unsupervised method for assessing transcriptomic gene-set enrichment. GSVA computes a comprehensive score for predefined gene sets by aggregating gene-level alterations to the pathway level to infer sample-specific biological functions. Gene sets for this investigation were sourced from the Molecular Signatures Database (MSigDB). The GSVA algorithm was subsequently applied to calculate enrichment scores for each set, facilitating the evaluation of potential changes in biological function across samples.

### 2.10 Weighted gene co-expression network analysis (WGCNA)

WGCNA was employed to discover co-expressed gene modules, investigate correlations between gene networks and disease phenotypes, and identify hub genes. A co-expression network for HMOX1 was created in the Kawasaki disease dataset using the WGCNA R package, focusing on the top 5000 genes by variance. A soft thresholding power value of 21 was used. The weighted adjacency matrix was transformed into a Topological Overlap Matrix (TOM) to assess network connectivity, and a hierarchical clustering dendrogram was generated from the TOM.

### 2.11 Drug prediction

The Drug-Gene Interaction Database (DGIdb) serves as a comprehensive repository of druggable genomes and drug-gene interactions (30). We utilized the DGIdb to identify potential drugs or molecular compounds that interact with the key genes. This database catalogs over 14,000 drug-gene interactions, encompassing 2,600 genes，6,300 drugs, and an additional 6,700 genes.

### 2.12 Molecular docking

Three-dimensional protein structures for the key genes were obtained from the AlphaFold database. Potential drugs targeting these genes, predicted via the DGIdb, had their structural data retrieved from PubChem. Molecular docking was performed in AutoDock using a genetic algorithm over 50 independent runs. The conformation exhibiting the lowest binding energy was selected for analysis. The resulting complexes were visualized in PyMOL to depict the binding sites between the small molecules and the target proteins.

### 2.13 Patient blood samples and ethical considerations

From March 2024 to March 2025, blood samples and clinical data were collected from 35 children with acute KD and 35 age-matched healthy controls at the Affiliated Hospital of Nantong University. All KD diagnoses adhered to the criteria established in the 2017 American Heart Association guidelines (4). None of the acute KD patients had received intravenous immunoglobulin (IVIG; 2 g/kg) or aspirin (30–50 mg/kg/day) before sampling. The healthy controls, confirmed to be free of infection or other illnesses through routine examination, served as the comparison group. All samples were immediately stored at −80 °C. Detailed clinical characteristics are provided in Supplementary Table S1. Written informed consent was obtained from participants or their guardians. This study was approved by the Ethics Committee of the Affiliated Hospital of Nantong University and conducted in accordance with the Declaration of Helsinki.

### 2.14 Isolation of peripheral blood mononuclear cells (PBMCs)

Peripheral blood mononuclear cells (PBMCs) were isolated from fresh anticoagulated blood using Ficoll density gradient centrifugation with lymphocyte separation medium (Axis-Shield, China). The isolated PBMCs were washed twice with phosphate-buffered saline.

### 2.15 Quantitative real-time polymerase chain reaction (RT-qPCR)

Total RNA was extracted from cells using TRIzol reagent (Invitrogen, USA). This RNA was then reverse-transcribed into cDNA using HiScript III RT SuperMix with a gDNA eraser (Vazyme). RT-qPCR was performed using SYBR Green (4368577, Thermo Scientific) with GAPDH as the endogenous control. All primer sequences are provided in Supplementary Table S2.

### 2.16 Western blot analysis

Cellular proteins were extracted using RIPA buffer supplemented with protease and phosphatase inhibitors. Protein concentrations were determined with a BCA assay kit (23225, Thermo Scientific, USA). Proteins were separated by 10% SDS-PAGE and transferred to PVDF membranes. After blocking with 5% non-fat milk for one hour, membranes were incubated with primary antibodies overnight at 4 °C. After three 5-minute washes with TBST, membranes were incubated with HRP-conjugated secondary antibodies for 1 h, then washed four times for 15 min each with TBST. Protein bands were visualized using an ECL solution and quantified with ImageJ software. The primary antibodies used were HMOX1 (ab13243, Abcam) and *β*-actin (4970, CST).

### 2.17 Animal model and experimental design

Male C57BL/6 mice aged 3 to 4 weeks were housed under specific pathogen-free (SPF) conditions at the Laboratory Animal Center of Nantong University. Following a one-week acclimatization period, the mice were randomly divided into three groups (*n* = 10 per group): a PBS control group, a CAWS group, and a CAWS + SnMP group. To model KD, mice in the CAWS and CAWS + SnMP groups received daily intraperitoneal injections of the Candida albicans water-soluble fraction (CAWS, 4 mg per mouse) for five consecutive days, as described previously (31, 32); the CAWS was prepared from Candida albicans strain NBRC1385. Control mice received an equivalent volume of PBS. Mice in the CAWS + SnMP group were additionally administered an intraperitoneal injection of SnMP (5 mg/kg) three weeks after the CAWS injections. Coronary artery dilation was evaluated by echocardiography on day 28. Subsequently, the mice were euthanized, and their hearts were harvested for analysis. All procedures complied with the National Institutes of Health guidelines and were approved by the Animal Ethics Committee [Approval Number: S20250607-009].

### 2.18 Immunohistochemical analysis

Mouse hearts were fixed in 4% paraformaldehyde for 24 h, embedded in paraffin, and sectioned at 5 µm. Sections were stained with hematoxylin and eosin (H&E) for histological assessment, and cardiac inflammation was scored according to established criteria (33). For immunohistochemistry, sections were deparaffinized and rehydrated. Antigen retrieval was performed by heating the sections in citrate buffer for 10 min. Endogenous peroxidase activity was quenched with 3% H₂O₂ for 10 min at room temperature, and non-specific binding was blocked with 3% bovine serum albumin (BSA) for 30 min. The sections were then incubated overnight at 4 °C with primary antibodies, followed by 1 h at room temperature with appropriate secondary antibodies. Signal detection used 3,3'-diaminobenzidine (DAB), and sections were counterstained with hematoxylin for 2 min, mounted with neutral resin, and examined under an optical microscope. Histopathological evaluation and vascular inflammation scoring were performed independently by three senior researchers, all blinded to group assignments.

### 2.19 Immunofluorescence (IF) staining

Cardiac tissues were fixed in 4% paraformaldehyde (PFA) at 4 °C for 24 h and embedded in paraffin. Sections (5 µm thick) were deparaffinized in xylene and rehydrated through a graded ethanol series. Antigen retrieval was conducted by heating the sections in sodium citrate buffer (10 mM sodium citrate, 0.05% Tween 20, pH 6.0) for 15 min. After permeabilization with 0.3% Triton X-100 for 30 min at room temperature and three washes with PBS, sections were blocked with 10% normal goat serum for 1 h. They were then incubated overnight at 4 °C with a primary antibody mixture against HMOX1 and F4/80 (ab6640, Abcam). Following PBS washes, sections were incubated for 1 h in the dark with a mixture of Alexa Fluor-conjugated secondary antibodies: goat anti-rabbit IgG Alexa Fluor 488 (A-11008, Invitrogen) and goat anti-rat IgG Alexa Fluor 594 (A-11007, Invitrogen). Finally, sections were washed, nuclei were counterstained with 4′,6-diamidino-2-phenylindole (DAPI) for 5 min, and slides were mounted with an anti-fade medium. Imaging was performed using a Leica fully automated inverted fluorescence microscope, and images were analyzed with ImageJ software.

### 2.20 High-resolution echocardiography in mice

Mice were anesthetized with isoflurane. Hair was removed from the left chest using depilatory cream, and ultrasound gel was applied. The diameter of the left main coronary artery (LCA) adjacent to the left sternum was measured using a high-resolution small animal ultrasound system (VEVO2100, FUJIFILM VisualSonics Inc., Toronto, ON, Canada).

### 2.21 Statistical analysis

All data are from at least three independent experiments, with biological and technical replicates performed in triplicate. Analyses were conducted using R software and GraphPad Prism version 9.0. Data are expressed as mean ± SEM. For two-group comparisons, an independent t-test was used for normally distributed data with equal variances and the Mann–Whitney U test for non-normal distributions. One-way ANOVA with Tukey's *post-hoc* tests was applied for multiple-group comparisons.Associations among variables were examined using Spearman's rank correlation analysis. A *p*-value of less than 0.05(*p* < 0.05)was considered statistically significant.

## 3 Results

### 3.1 Quality control and data normalization

Based on the quality assessment of multiple samples, cells with fewer than 200 captured genes and abnormal values will be filtered. Subsequently, the DoubletFinder package is used to filter for double cells, retaining 73,933 cells—violin and scatter plots after filtering (Supplementary Figure S1A, B). Subsequently, we identified 2,000 variable genes (Supplementary Figure S1C) and then performed normalization, PCA, and harmony analysis on the data (Supplementary Figure S1D–F).

### 3.2 Single-cell atlas of circulating immune cells in KD

**Figure 1:**
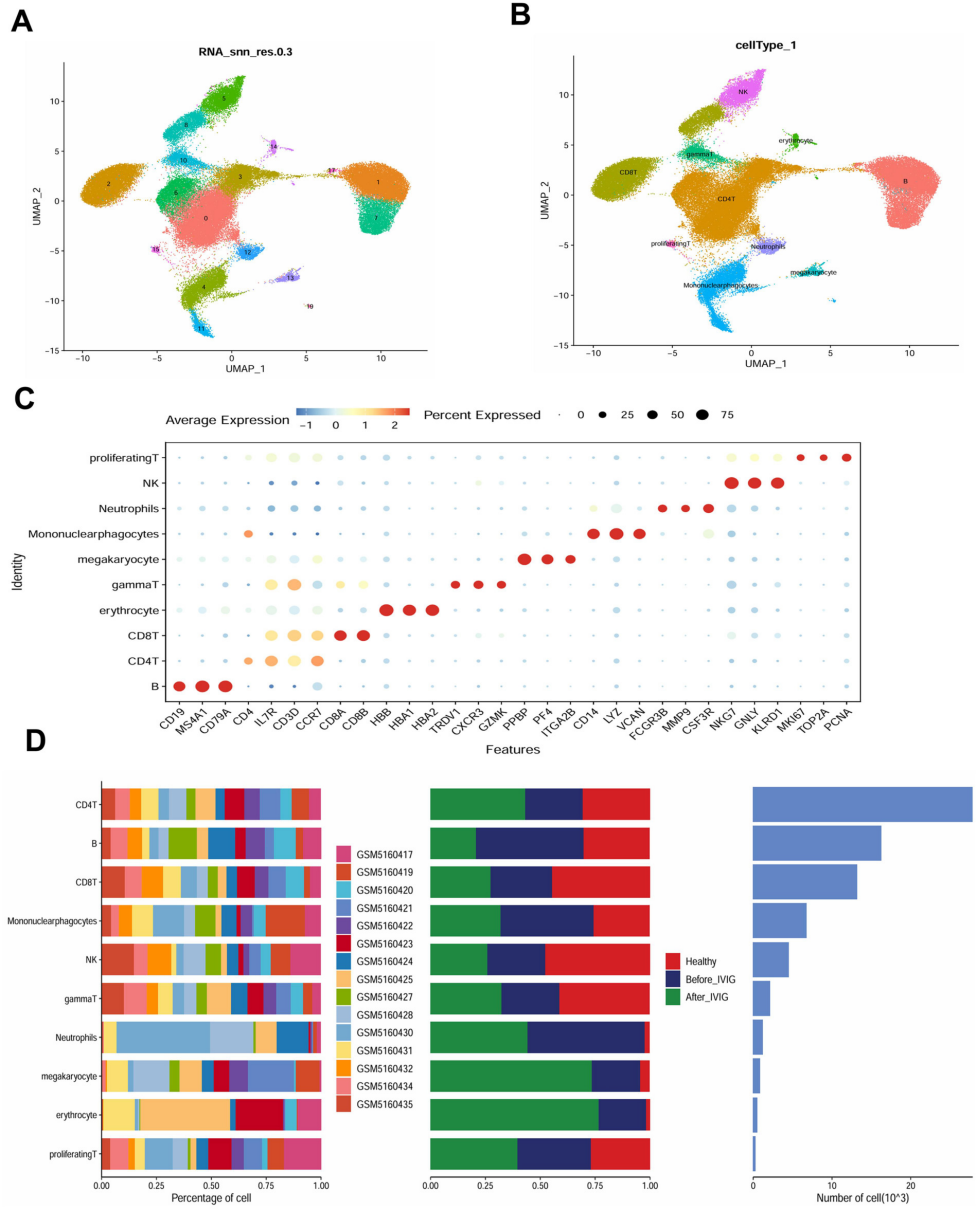
Single-cell atlas of circulating immune cells in KD and healthy controls. **(A)** A Uniform Manifold Approximation and Projection (UMAP) plot depicts the distinct cell clusters identified through unsupervised clustering of the integrated dataset, with each color representing an independent cluster. **(B)** The same UMAP plot is shown, now colored by cell type as annotated using canonical marker gene expression. **(C)** A dot plot visualizes the expression of canonical marker genes across the major cell types; dot size indicates the percentage of cells expressing the gene within a cluster, and color intensity reflects the average expression level in those cells. **(D)** Bar charts present the relative proportions and absolute cell numbers for each major cell type across all samples and groups.

UMAP analysis identified seventeen distinct clusters (Figure 1A). Peripheral blood mononuclear cells (PBMCs) from the samples were processed for single-cell RNA sequencing (scRNA-seq) on the 10× Genomics platform. After quality control, 73,933 cells remained for analysis: 28,844 from patients before intravenous immunoglobulin (IVIG) treatment, 30,599 from patients after treatment, and 14,490 from healthy controls. We annotated the clusters as T cells (expressing CD3D, CD3E, CD3G), CD4+ T cells (CD4), CD8+ T cells (CD8A, CD8B), natural killer (NK) cells (NKG7, GNLY), B cells (MS4A1, TCL1A, TNFRSF13B), and mononuclear phagocytes (CD14, LYZ). The PBMC samples also contained a small number of residual erythrocytes (HBB, HBA1) and megakaryocytes (PPBP, PF4) (Figure 1B). Subsequently, a systematic analysis of myeloid cell subpopulations in the single-cell RNA sequencing data was performed. UMAP-based dimensionality reduction and clustering showed that myeloid cells could be divided into five distinct subpopulations, including cDC2, classical monocytes, IFN-stimulated monocytes, non-classical monocytes, and plasmacytoid dendritic cells. (Supplementary Figure S2A, B). A bubble plot visualizes the expression of these canonical markers across cell types (Figure 1C). A bar chart comparing the proportional distribution of cell types between groups showed that, relative to healthy controls, pre-treatment KD patients had a higher percentage of mononuclear phagocytes and B cells (Figure 1D).

### 3.3 Expression characteristics of HMOX1 at the single-cell level and its regulatory role in intercellular communication

**Figure 2:**
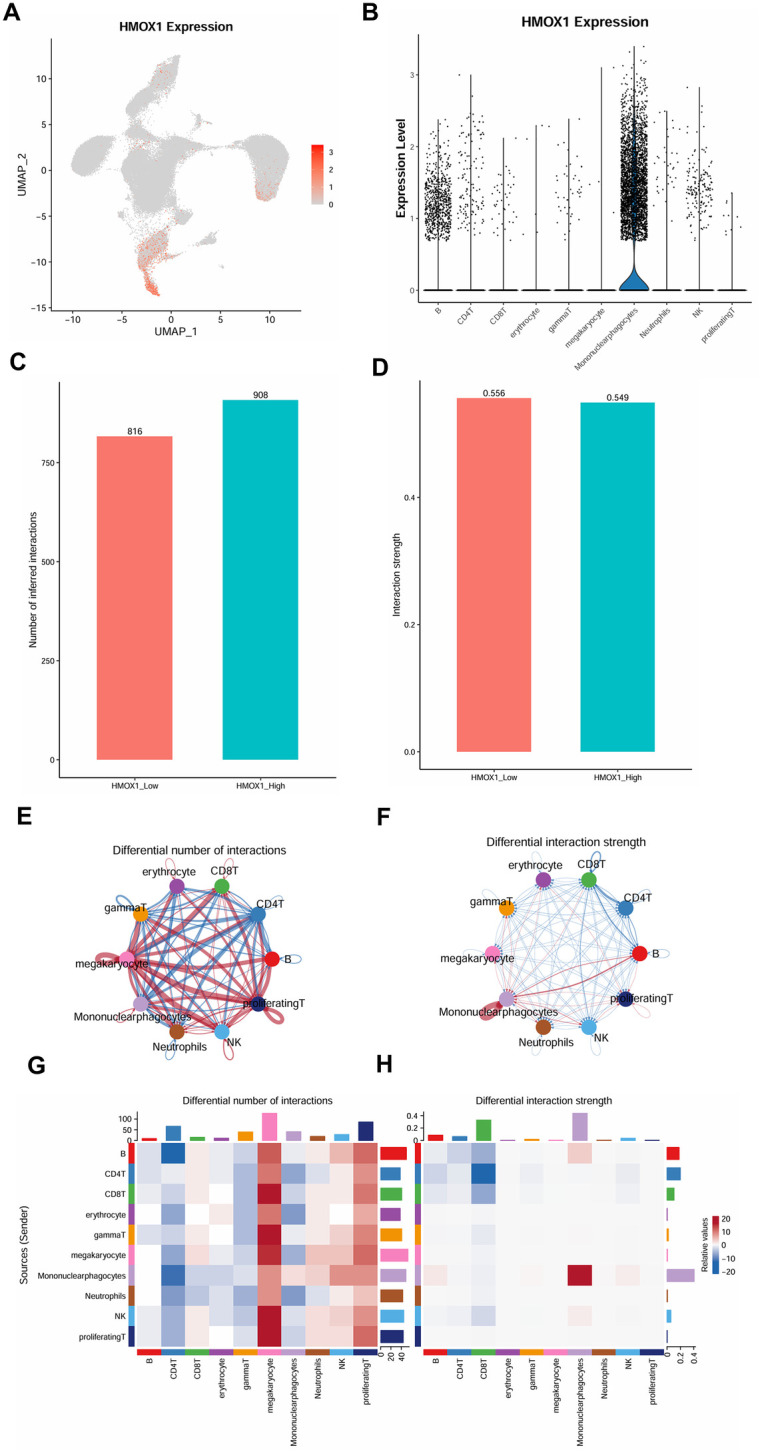
Expression characteristics of HMOX1 at the single-cell level and its regulatory role in intercellular communication. **(A)** A feature plot illustrates the single-cell transcriptome expression profile of HMOX1 on a UMAP projection, employing a color gradient from gray to red to signify relative expression levels. **(B)** A violin plot illustrates the distribution of HMOX1 expression among several cell types, demonstrating significant cell type selectivity; mononuclear phagocytes display the greatest expression levels. **(C)** Bar charts illustrate the comparative cellular communication between HMOX1-high and HMOX1-low groups, quantifying the total number (left) and total strength (right) of interactions. **(D)** A Circos graphic displays a differential network topology study, depicting alterations in the quantity (left) and intensity (right) of ligand-receptor interactions. **(E,F)** Circos plots illustrating the differential cell-cell communication networks between the two groups. The left panel represents changes in the number of communication events, while the right panel represents changes in communication strength. **(G,H)** Heatmaps showing the differences in the number and strength of cell-cell communications between the two groups. The left panel displays differences in the number of communications, and the right panel displays differences in communication strength. The vertical axis represents the ligand cell types, and the horizontal axis represents the receptor cell types.

We visualized the expression profile of HMOX1 across single cells using the FeaturePlot and VlnPlot functions from the Seurat R package (Figure 2A). HMOX1 expression showed notable cell-type specificity, with the highest levels detected in mononuclear phagocytes (Figure 2B). Furthermore, we investigated the expression of HMOX1 across distinct subpopulations of mononuclear phagocytes. Violin plot analysis revealed that non-classical monocytes exhibited the highest expression level of HMOX1, along with the broadest expression distribution and the most pronounced intercellular heterogeneity. These findings suggest that this subpopulation may be subjected to enhanced oxidative stress or exhibit more active heme metabolism. The cDC2 subpopulation also displayed a moderate level of HMOX1 expression, albeit lower than that observed in non-classical monocytes. In contrast, classical monocytes and IFN monocytes demonstrated relatively low HMOX1 expression, while pDCs showed nearly undetectable levels of HMOX1 expression, with values approaching zero (Supplementary Figure S3A). To investigate how high HMOX1 expression affects immune cell interactions, we stratified cells into HMOX1_high and HMOX1_low groups based on single-cell expression data and compared their overall cell communication networks. The HMOX1_high group exhibited a significantly greater number of intercellular interactions than the HMOX1_low group, although the overall communication strength remained largely unchanged (Figures 2C,D). This implies that elevated HMOX1 expression may expand the repertoire of communication pathways rather than intensify individual signaling events. Analysis of specific cell-pair interactions identified particularly close communication links between mononuclear phagocytes and B cells (Figures 2E,F). A systematic comparison of communication differences further showed that high HMOX1 expression markedly altered the number of interactions between mononuclear phagocytes and other immune cells. Notably, interactions with neutrophils, monocytes, and certain T-cell subsets were strengthened (Figures 2G,H). These results indicate that HMOX1, beyond its specific high expression in mononuclear phagocytes, may be closely linked to the remodeling of the intercellular communication network in the peripheral blood immune microenvironment of KD. To further elucidate the molecular basis of the cell–cell communication network remodeling associated with high *HMOX1* expression, we performed CellChat bubble plot analyses comparing ligand–receptor interactions involving mononuclear phagocytes between the *HMOX1*-high and *HMOX1*-low groups. When mononuclear phagocytes were analyzed as signaling sources, multiple ligand–receptor pairs showed increased predicted communication probabilities in the *HMOX1*-high group, including ANXA1–FPR1, LGALS9–PTPRC/CD45, LGALS9–CD44, MIF–CD74/CXCR4, ICAM1/ICAM2–integrin complexes, CD99–PILRA, and HLA-related interactions (Supplementary Figure S4A). Similar increases were observed when mononuclear phagocytes were analyzed as signaling targets, including ANXA1–FPR1, LGALS9–PTPRC/CD45, LGALS9–CD44, ICAM1/ICAM2–integrin complexes, HLA class II–CD4 interactions, and CD99–PILRA (Supplementary Figure S4B). These findings suggest that *HMOX1*-high mononuclear phagocytes exhibit a more active and diversified intercellular communication pattern involving inflammatory regulation, antigen presentation, leukocyte adhesion, and immune-cell crosstalk in the inflammatory microenvironment of KD. Future studies are warranted to further validate and mechanistically characterize these inferred ligand–receptor interactions.

### 3.4 Quality control and differential expression analysis of integrated KD data

Raw gene expression profiles for KD were obtained from the Gene Expression Omnibus (GEO) and consolidated across datasets. Principal component analysis (PCA) was used to evaluate global expression patterns before and after batch correction. Before adjustment, samples were distinctly grouped by batch, indicating a significant batch impact. Following batch adjustment using the ComBat approach, the separation induced by batch effects was significantly reduced, resulting in an intermixed distribution of samples across batches, indicating efficient removal of batch-related variance (Supplementary Figure S5A, B). The patient cohort showed elevated HMOX1 expression compared with the control cohort, indicating increased HMOX1 in the disease state (Supplementary Figure S5C). A study of HMOX1 expression between healthy controls and patients revealed a statistically significant difference (*p* < 0.05). The results suggest that HMOX1 may be involved in the initiation and course of disease, possessing potential biological activities and clinical significance in KD.

### 3.5 Correlation between HMOX1 expression and immune cell infiltration

We employed the CIBERSORT algorithm to quantify infiltration of 22 immune cell types to investigate the role of HMOX1 in KD immunology. A stacked bar chart was created to depict significant immunological variability within the KD microenvironment (Supplementary Figure S6A). A correlation heatmap elucidated a complex network of positive and negative relationships among immune cell types, suggesting potential synergistic and antagonistic interactions (Supplementary Figure S6B). A comparison of immunological profiles between high- and low-HMOX1 expression groups revealed substantial differences in the infiltration of specific immune cells (Supplementary Figure S6C). Monocytes, activated dendritic cells, and M0 mononuclear phagocytes were markedly enriched in the high-expression group, suggesting that increased HMOX1 was associated with a specific pattern of innate immune activation. Subsequently, we conducted a correlation study between HMOX1 expression levels and the proportion of immune-cell infiltration (Supplementary Figure S6D).HMOX1 expression showed a strong positive association with pro-inflammatory cell populations, including monocytes and neutrophils, while demonstrating a substantial negative correlation with immunoregulatory populations, such as M2 mononuclear phagocytes and resting mast cells. The data suggest that HMOX1 may be crucial in altering the immune cell composition during the KD inflammatory response.

### 3.6 Signaling pathways involving the HMOX1

**Figure 3:**
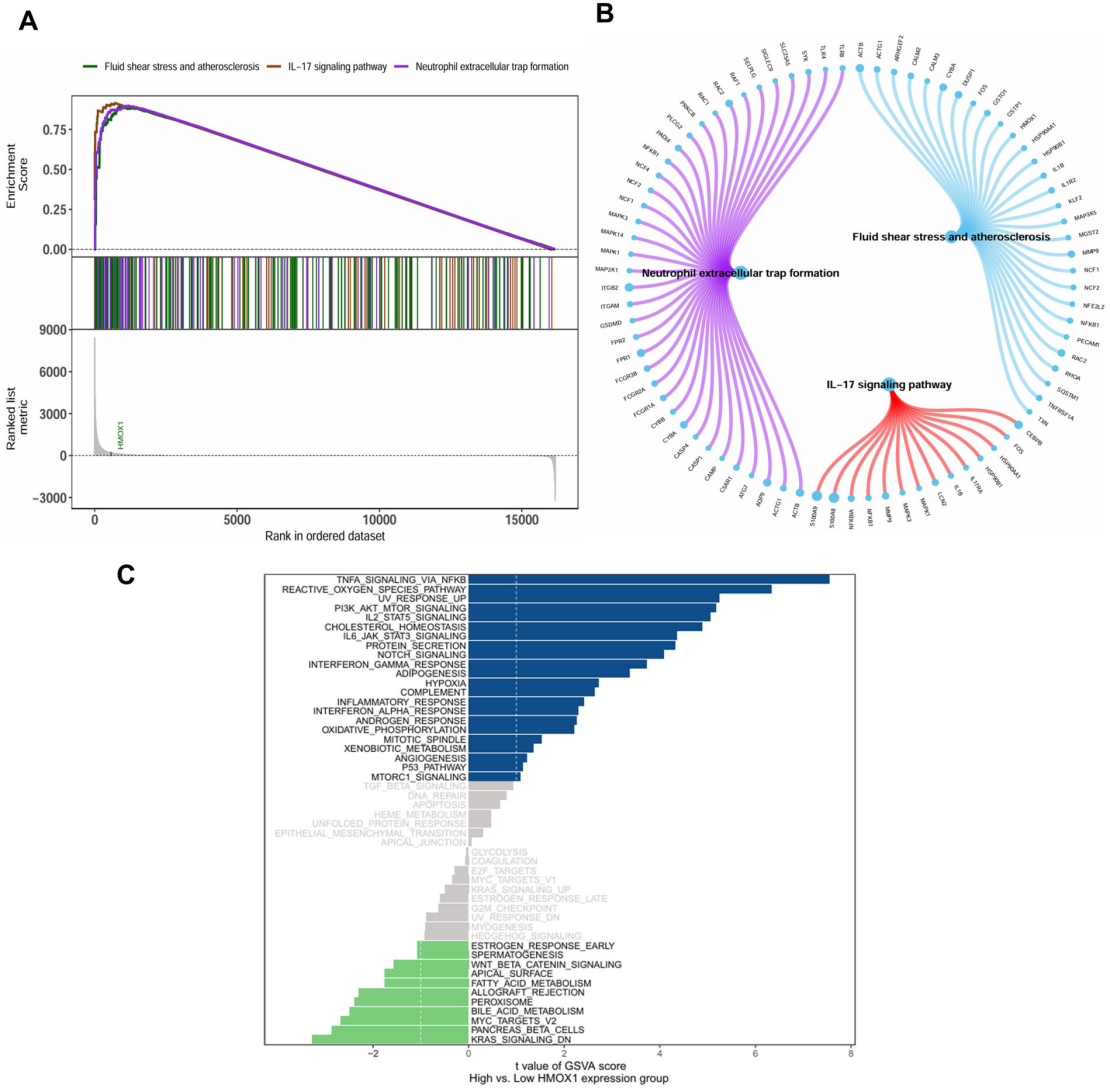
GSVA and GSEA analyses. **(A)** GSEA of the entire genome transcriptome, utilizing HMOX1 expression groups as phenotypic labels, produced these enrichment plots. The *x*-axis displays genes ordered by their link with the phenotype, and the *y*-axis represents the enrichment score (ES). Pathways that are significantly and positively enriched (NES > 0, adjusted *P* < 0.05) in the high HMOX1 expression group include “Fluid shear stress and atherosclerosis,” “IL-17 signaling pathway,” and “Neutrophil extracellular trap formation.” **(B)** A Circos chord diagram illustrates the overlap of conserved genes across strongly enriched GSEA pathways, highlighting core, common genes and their interrelationships. **(C)** This bar chart illustrates the outcomes of the GSVA algorithm, emphasizing signaling pathways with notable activity disparities (t-values) between the high- and low-expression groups of HMOX1.

To examine the potential molecular mechanisms by which HMOX1 affects illness progression, we analyzed its related signaling pathways. We conducted multidimensional functional enrichment analyses using two complementary methodologies: GSEA and GSVA.GSEA indicated that, in contrast to the low HMOX1 expression group, the high-expression group exhibited significant positive enrichment in inflammation- and vasculature-associated pathways, such as the IL-17 signaling pathway and neutrophil extracellular trap formation (NES > 0, adjusted *P* < 0.05; Figure 3A). A Circos plot of gene-pathway interactions was created to clarify the inherent relationships among these pathways, illustrating significant gene sharing and functional intercommunication (Figure 3B).GSVA additionally suggested that individuals with elevated HMOX1 expression showed significant upregulation of canonical pro-inflammatory and oxidative stress-related pathways, including TNFA_SIGNALING_VIA_NFKB, REACTIVE_OXYGEN_SPECIES_PATHWAY, IL2_STAT5_SIGNALING, and PROTEIN_SECRETION.In contrast, pathways associated with metabolism and homeostasis, such as fatty acid metabolism, bile acid metabolism, and peroxisome functions, were comparatively enriched in the group with low HMOX1 expression (Figure 3C). These findings indicate that HMOX1 may be pivotal in the vascular damage mechanism of KD by mediating the NF-*κ*B inflammatory cascade and modulating oxidative stress.

### 3.7 Identification of HMOX1-associated co-expression modules and their potential immune regulatory mechanisms based on WGCNA

GSEA and GSVA demonstrated a robust correlation between HMOX1 expression and inflammatory and vascular disease; nevertheless, gene functions generally function within intricate co-expression networks. To elucidate the prospective regulatory network associated with HMOX1 from a systems viewpoint, we conducted WGCNA on whole-transcriptome data from patients with KD. Employing a soft-thresholding power (*β*) of 21 (Supplementary Figure S7A, B), module-trait connection analysis revealed the yellow module as the most significantly correlated with HMOX1 (co*r* = 0.33, *p* = 9e−08), indicating a strong functional association (Supplementary Figure S7C). A study of the yellow module genes was performed to elucidate their biological functions. Significantly elevated Gene Ontology terms encompassed positive modulation of defense response, secretory granule membrane, and actin binding, aligning with the characteristics of immune cell activation and migration in KD (Supplementary Figure S7D, E). Furthermore, KEGG pathway analysis underscored the centrality of this HMOX1-associated network, revealing significant enrichment in Salmonella infection, Osteoclast differentiation, Tuberculosis, and Lipid and atherosclerosis pathways, which clarify its important link to inflammatory and vascular pathology (Supplementary Figure S7F, G).

### 3.8 Identification of potential therapeutic agents targeting HMOX1 and molecular docking analysis

**Figure 4:**
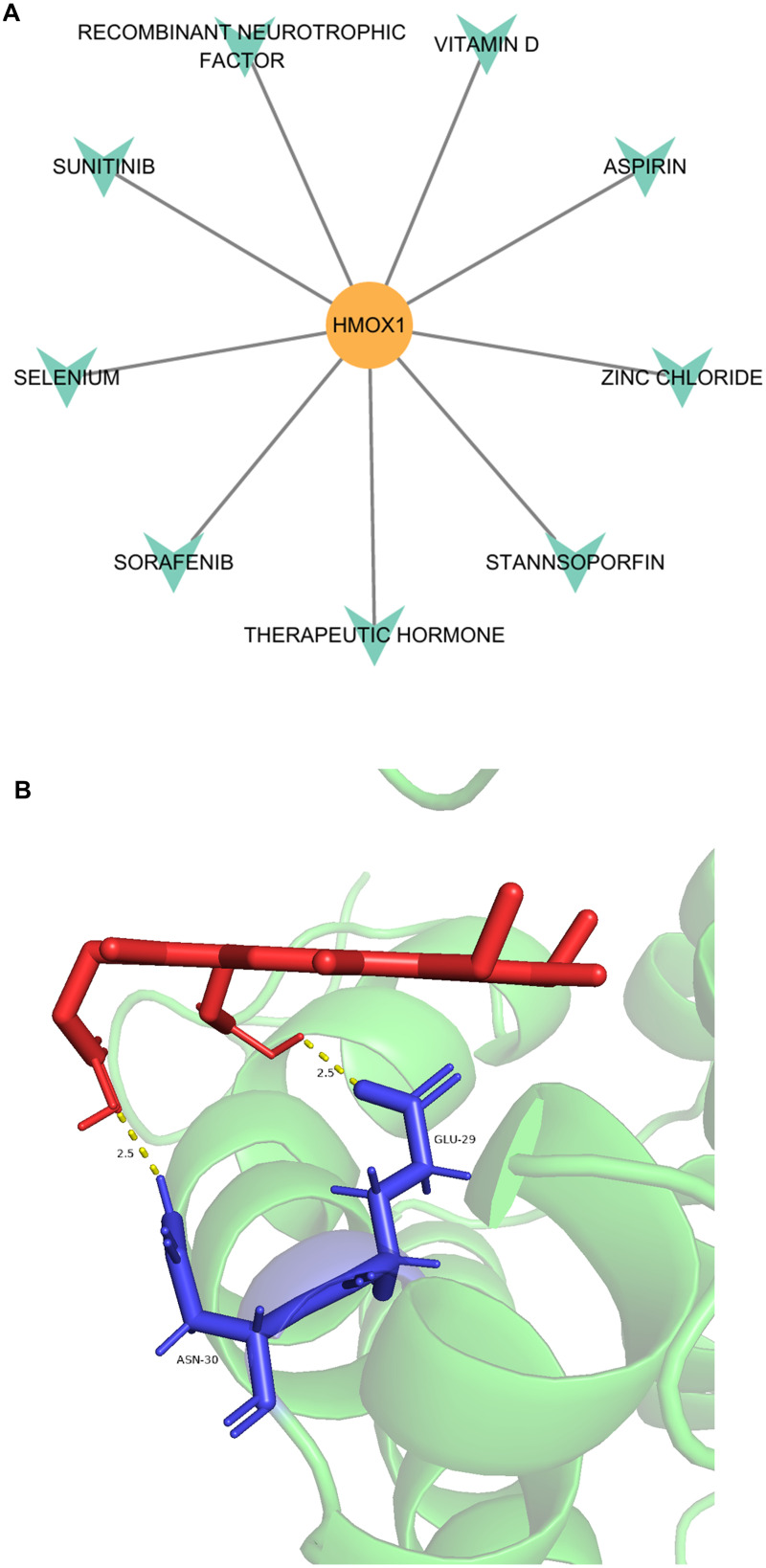
Identification of potential therapeutic agents targeting HMOX1 and molecular docking analysis. **(A)** The drug-gene interaction network for HMOX1 was predicted using the DGIdb database. **(B)** This network illustrates the interactions between HMOX1 and various molecules. Each node represents a molecule, and the connecting lines denote molecular docking or other interactions.

A drug-gene interaction network was constructed using the DGIdb database to explore the clinical translational potential of HMOX1 and identify potential targeted interventions. This screening identified nine drugs and small-molecule compounds with known or possible interactions with HMOX1 (Figure 4A), including anti-inflammatory and immunomodulatory agents (e.g., Aspirin, Vitamin D, Selenium), kinase inhibitors (e.g., Sunitinib, Sorafenib), and metalloporphyrin compounds (e.g., Stannsoporfin). For subsequent functional validation of HMOX1, Stannsoporfin was selected for further study. As a metalloporphyrin derivative, Stannsoporfin is a competitive inhibitor of HMOX1 (34). Molecular docking simulations were performed to confirm a direct interaction, demonstrating that Stannsoporfin binds stably within the active site pocket of HMOX1 (P09601) with a binding energy of −5.7 kcal/mol (Figure 4B). Structural analysis revealed that Stannsoporfin forms key hydrogen bonds (bond length ∼2.5 Å) with the GLU-29 and ASN-30 residues in the protein pocket, which stabilizes the HMOX1-Stannsoporfin complex. This molecular-level evidence establishes Stannsoporfin as an ideal tool compound for targeting HMOX1, providing a theoretical basis for its use in subsequent *in vivo* functional validation studies.

### 3.9 Expression levels of HMOX1 and TNF-α in clinical samples and KD mouse models

**Figure 5:**
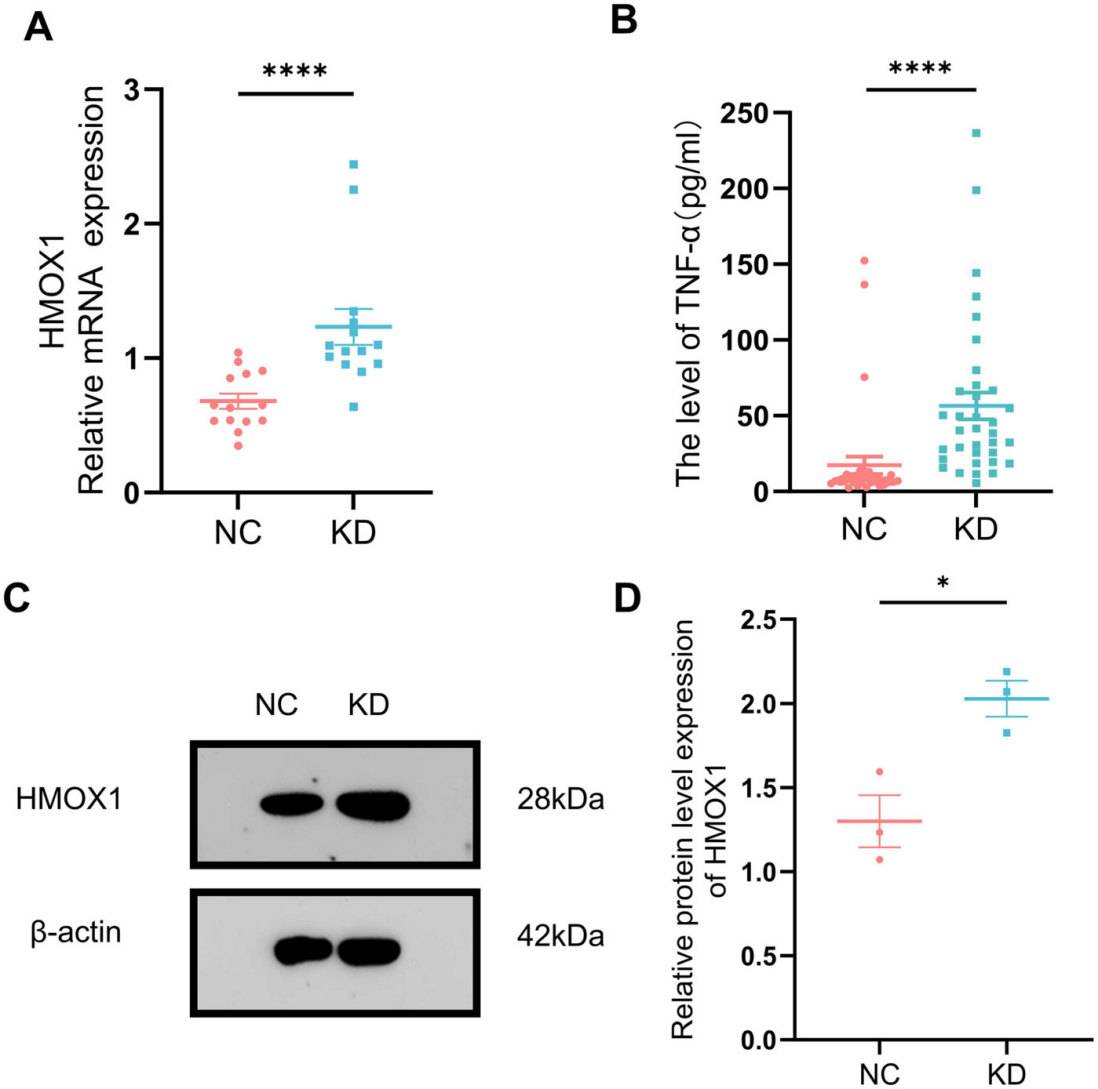
Upregulated expression of HMOX1 and TNF-α in patients with KD and KD mouse models. **(A)** qRT-PCR compared the relative mRNA expression of HMOX1 in peripheral blood mononuclear cells (PBMCs) from KD patients and healthy controls. **(B)** Serum concentrations of tumor necrosis factor-α were measured by enzyme-linked immunosorbent assay in KD patients and healthy controls. **(C)** Representative western blot showing HMOX1 protein expression in cardiac tissues from control mice and a KD mouse model, with *β*-actin as a loading control. **(D)** Densitometric quantification of the western blot results is shown (*n* = 3 per group). These represent cardiac tissues from three different mice. Data are expressed as mean ± SEM. Figures 5A, 5B were analyzed using the Mann–Whitney U test, and Figure 5D was analyzed using the independent-samples t-test. *p < 0.05, ****p < 0.0001.

Quantitative real-time polymerase chain reaction analysis of PBMCs showed that HMOX1 levels were significantly higher in KD patients than in the control group (Figure 5A). Serum levels of tumor necrosis factor-alpha, measured by enzyme-linked immunosorbent assay, were also markedly elevated in KD patients (Figure 5B). Furthermore, Western blot analysis confirmed higher HMOX1 expression in the KD mouse group compared to controls (Figures 5C,D).

### 3.10 SnMP ameliorates vascular inflammation in a murine model of KD vasculitis induced by HMOX1 in mononuclear phagocytes

**Figure 6:**
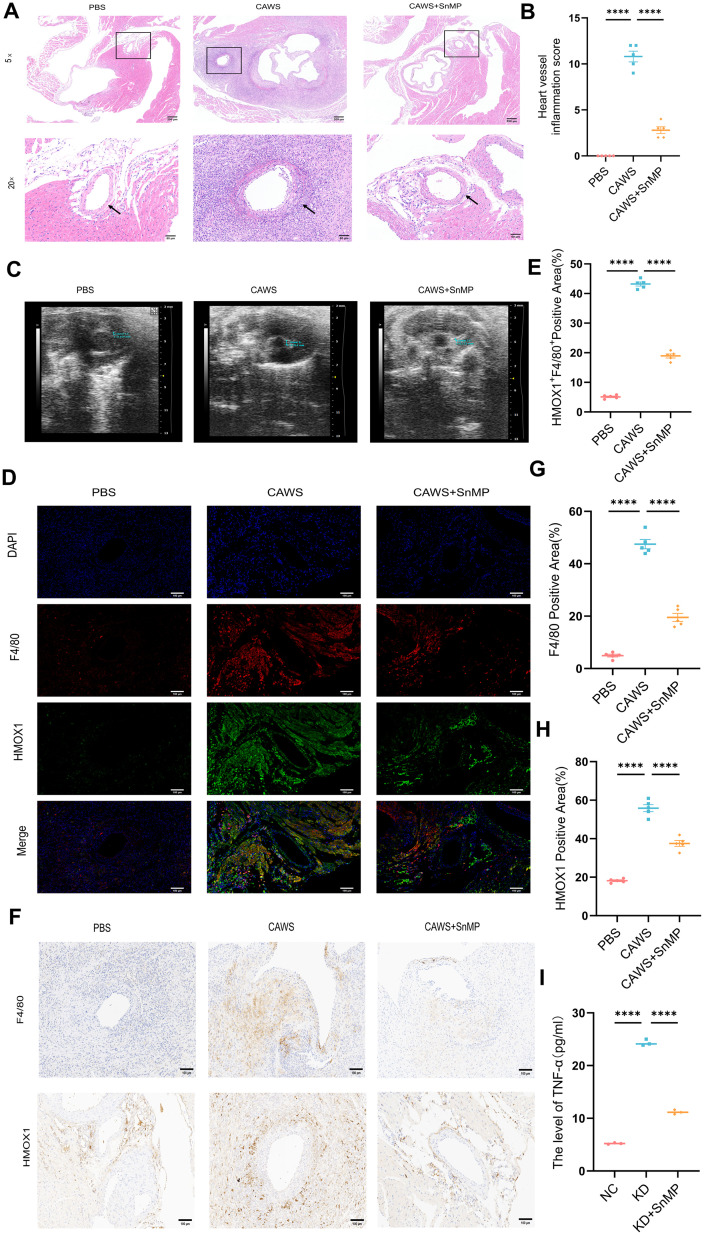
SnMP attenuates coronary artery inflammation and dilation in KD mouse models. **(A)** Representative H&E-stained sections of coronary arteries from each treatment group in the KD vasculitis model (*n* = 5 mice per group).CAWS administration triggered a pronounced inflammatory response with substantial immune cell infiltration in the vessel wall, which was markedly attenuated by treatment with the HMOX1 inhibitor SnMP. Scale ba*r* = 100 μm. **(B)** Quantitative assessment of cardiac vascular inflammation scores across groups. **(C)** Echocardiographic analysis of coronary artery internal diameter (*n* = 5 mice per group). CAWS induced significant coronary artery dilation, an effect that was substantially mitigated by SnMP treatment. Data are presented as mean ± SEM. **(D)** Representative immunofluorescence images depicting HMOX1 expression co-localized with F4/80 in the coronary artery lesion area (*n* = 5 mice per group). Scale ba*r* = 100 μm. **(E)** Quantitative analysis of HMOX1 and F4/80 co-localization from immunofluorescence staining. **(F)** Immunohistochemical staining of coronary artery tissue for F4/80 and HMOX1 expression. **(G,H)** Quantification of F4/80 and HMOX1 expression from the immunohistochemical analysis shown in **(F) (I)** Serum TNF-α levels measured by ELISA across groups. Data are expressed as mean ± SEM. Statistical significance was determined using one-way ANOVA followed by Tukey's *post hoc* test.****p < 0.0001.

To investigate the role of HMOX1 in KD, we induced cardiovascular inflammation in mice using the water-soluble fraction of Candida albicans(CAWS) to establish a KD model.CAWS administration triggered significant cardiovascular inflammation and coronary artery dilation. Treatment with the HMOX1 inhibitor tin mesoporphyrin (SnMP) markedly attenuated both coronary artery inflammation and dilation (Figures 6A–C). SnMP treatment also significantly reduced HMOX1 expression in macrophages**,** macrophage infiltration, and tumor necrosis factor-alpha secretion (Figures 6D–I).

## 4 Discussion

In this study, we found that HMOX1 was preferentially enriched in mononuclear phagocytes in acute KD and was associated with inflammatory and oxidative stress-related programs. Clinical validation showed increased HMOX1 expression in peripheral blood mononuclear cells and elevated serum tumor necrosis factor-alpha levels in patients with KD. These peripheral blood findings suggest that mononuclear phagocytes may represent an inflammatory circulating precursor state that is primed for migration into vascular tissues and subsequent macrophage-lineage differentiation. In the CAWS-induced murine model, increased HMOX1 expression was observed in macrophages infiltrating the coronary artery roots, and pharmacologic inhibition of heme oxygenase attenuated vascular inflammation and coronary artery injury. In the present study, SnMP was administered 3 weeks after CAWS induction. We selected this time point because vascular inflammation in the CAWS model remains active at this stage, while coronary and perivascular inflammatory lesions have already been established. Therefore, this intervention is better interpreted as targeting ongoing vasculitis and evolving coronary lesions rather than the earliest initiating phase of mononuclear phagocyte activation. Together, these findings suggest that increased HMOX1 is associated with a proinflammatory mononuclear phagocyte state in KD.

HMOX1 is classically regarded as a stress-inducible cytoprotective enzyme, but its biological effects are highly context dependent. Previous studies have shown that HMOX1 and its downstream metabolites, including carbon monoxide, biliverdin, and bilirubin, can exert antioxidant and anti-inflammatory effects in various inflammatory settings (35, 36). However, under conditions of excessive heme turnover, iron overload, or oxidative stress, increased heme oxygenase activity may promote reactive oxygen species generation and facilitate inflammatory signaling (37, 38). Reactive oxygen species, in turn, can activate the nuclear factor kappa B axis through various mechanisms, driving the transcription of pro-inflammatory genes such as tumor necrosis factor-alpha (39, 40). On the basis of our transcriptomic analyses and *in vivo* observations, we propose a working model in which increased HMOX1 in mononuclear phagocytes is associated with activation of oxidative stress-related pathways and the tumor necrosis factor-alpha/nuclear factor kappa B axis in KD.

This study has several limitations. First, the human transcriptomic and clinical data are cross-sectional and therefore cannot establish causality. Second, the whole-blood transcriptomic cohorts provide indirect estimates of immune cell composition rather than direct cell-specific functional evidence. Third, tin mesoporphyrin was used as a pharmacologic heme oxygenase inhibitor, and its effects should not be interpreted as definitive proof of HMOX1-specific causality. Fourth, labile iron pools, reactive oxygen species flux, and nuclear factor kappa B nuclear translocation were not directly measured. Fifth, HMOX1 expression in our independent clinical validation cohort was assessed only during the acute pre-IVIG phase. Although the public scRNA-seq dataset GSE168732 included KD samples obtained both before and after IVIG treatment, our clinical validation cohort did not include paired post-IVIG blood samples. Therefore, we were unable to directly validate whether HMOX1 expression changes after IVIG treatment in these patients. Future longitudinal studies using paired pre- and post-IVIG samples are needed to determine whether HMOX1 expression is modulated by IVIG treatment and whether such changes are associated with inflammatory resolution or treatment response. In addition, further cell-specific and mechanistic studies are needed to clarify the role of HMOX1 in mononuclear phagocyte activation and vascular inflammation in KD.

## 5 Conclusions

Multi-omics analyses, clinical validation, and *in vivo* experiments suggest that HMOX1 is preferentially upregulated in mononuclear phagocytes in acute Kawasaki disease and is associated with inflammatory and oxidative stress-related signaling. In a CAWS-induced murine vasculitis model, pharmacologic inhibition of heme oxygenase attenuated ongoing vascular inflammation and coronary artery injury, providing *in vivo* evidence that the heme oxygenase pathway may contribute to KD-related vascular inflammation and may represent a potential therapeutic target worthy of further investigation in KD.

## Data Availability

The original contributions presented in the study are publicly available. The KD-related datasets analyzed in this study are publicly available and searchable in the NCBI Gene Expression Omnibus (GEO) database under accession numbers GSE168732, GSE73461, and GSE68004.

## References

[1] Newburger JW Takahashi M Burns JC. Kawasaki disease. J Am Coll Cardiol. (2016) 67:1738–49. 10.1016/j.jacc.2015.12.073 27056781

[2] Noval Rivas M Arditi M. Kawasaki disease: pathophysiology and insights from mouse models. Nat Rev Rheumatol. (2020) 16:391–405. 10.1038/s41584-020-0426-0 32457494 PMC7250272

[3] Xiong Y Xu J Zhang D Wu S Li Z Zhang J. MicroRNAs in kawasaki disease: an update on diagnosis, therapy and monitoring. Front Immunol. (2022) 13:1016575. 10.3389/fimmu.2022.1016575 36353615 PMC9638168

[4] McCrindle BW Rowley AH Newburger JW Burns JC Bolger AF Gewitz M. Diagnosis, treatment, and long-term management of Kawasaki disease: a scientific statement for health professionals from the American Heart Association. Circulation. (2017) 135:e927–99. 10.1161/CIR.0000000000000484 28356445

[5] Tremoulet AH Best BM Song S Wang S Corinaldesi E Eichenfield JR. Resistance to intravenous immunoglobulin in children with kawasaki disease. J Pediatr. (2008) 153:117–21. 10.1016/j.jpeds.2007.12.021 18571548 PMC2526555

[6] Xie Z Huang Y Li X Lun Y Li X He Y. Atlas of circulating immune cells in kawasaki disease. Int Immunopharmacol. (2022) 102:108396. 10.1016/j.intimp.2021.108396 34890998

[7] Hara T Nakashima Y Sakai Y Nishio H Motomura Y Yamasaki S. Kawasaki disease: a matter of innate immunity. Clin Exp Immunol. (2016) 186:134–43. 10.1111/cei.12832 27342882 PMC5054572

[8] Sun H Jiang J Gong L Li X Yang Y Luo Y. Voltage-gated sodium channel inhibitor reduces atherosclerosis by modulating monocyte/macrophage subsets and suppressing macrophage proliferation. Biomed Pharmacother. (2019) 120:109352. 10.1016/j.biopha.2019.109352 31586905

[9] Liao P Tong S Du L Mei J Wang B Lu Y. Single-cell transcriptomics identifies the common perturbations of monocyte/macrophage lineage cells in inflammaging of bone marrow. J Orthop Translat. (2025) 50:85–96. 10.1016/j.jot.2024.09.013 39868348 PMC11762928

[10] Mehta H Mashiko S Angsana J Rubio M Hsieh YM Maari C. Differential changes in inflammatory mononuclear phagocyte and T-cell profiles within psoriatic skin during treatment with guselkumab vs. Secukinumab. J Invest Dermatol. (2021) 141:1707–1718.e9. 10.1016/j.jid.2021.01.005 33524368

[11] Liu W Zhang Y Zhu W Ma C Ruan J Long H. Sinomenine inhibits the progression of rheumatoid arthritis by regulating the secretion of inflammatory cytokines and monocyte/macrophage subsets. Front Immunol. (2018) 9:2228. 10.3389/fimmu.2018.02228 30319663 PMC6168735

[12] Hume DA. The mononuclear phagocyte system. Curr Opin Immunol. (2006) 18:49–53. 10.1016/j.coi.2005.11.008 16338128

[13] Banchereau R Cepika AM Banchereau J Pascual V. Understanding human autoimmunity and autoinflammation through transcriptomics. Annu Rev Immunol. (2017) 35:337–70. 10.1146/annurev-immunol-051116-052225 28142321 PMC5937945

[14] Tenhunen R Marver HS Schmid R. The enzymatic conversion of heme to bilirubin by microsomal heme oxygenase. Proc Natl Acad Sci U S A. (1968) 61:748–55. 10.1073/pnas.61.2.748 4386763 PMC225223

[15] Ryter SW Choi AM. Heme oxygenase-1/carbon monoxide: from metabolism to molecular therapy. Am J Respir Cell Mol Biol. (2009) 41:251–60. 10.1165/rcmb.2009-0170TR 19617398 PMC2742746

[16] Liu R Zhang X Nie L Sun S Liu J Chen H. Heme oxygenase 1 in erythropoiesis: an important regulator beyond catalyzing heme catabolism. Ann Hematol. (2023) 102:1323–32. 10.1007/s00277-023-05193-7 37046065

[17] Otterbein LE Bach FH Alam J Soares M Tao Lu H Wysk M. Carbon monoxide has anti-inflammatory effects involving the mitogen-activated protein kinase pathway. Nat Med. (2000) 6:422–8. 10.1038/74680 10742149

[18] Brouard S Otterbein LE Anrather J Tobiasch E Bach FH Choi AM. Carbon monoxide generated by heme oxygenase 1 suppresses endothelial cell apoptosis. J Exp Med. (2000) 192:1015–26. 10.1084/jem.192.7.1015 11015442 PMC2193315

[19] Siow RC Sato H Mann GE. Heme oxygenase-carbon monoxide signalling pathway in atherosclerosis: anti-atherogenic actions of bilirubin and carbon monoxide? Cardiovasc Res. (1999) 41:385–94. 10.1016/S0008-6363(98)00278-8 10341838

[20] Ryter SW Tyrrell RM. The heme synthesis and degradation pathways: role in oxidant sensitivity: heme oxygenase has both pro- and antioxidant properties. Free Radic Biol Med. (2000) 28:289–309. 10.1016/S0891-5849(99)00223-3 11281297

[21] Peng X Sun B Tang C Shi C Xie X Wang X. HMOX1-LDHB Interaction promotes ferroptosis by inducing mitochondrial dysfunction in foamy macrophages during advanced atherosclerosis. Dev Cell. (2025) 60:1070–1086.e8. 10.1016/j.devcel.2024.12.011 39731912

[22] Morgan MJ Liu ZG. Crosstalk of reactive oxygen species and NF-*κ*B signaling. Cell Res. (2011) 21:103–15. 10.1038/cr.2010.178 21187859 PMC3193400

[23] Vijayan V Wagener FADTG Immenschuh S. The macrophage heme-heme oxygenase-1 system and its role in inflammation. Biochem Pharmacol. (2018) 153:159–67. 10.1016/j.bcp.2018.02.010 29452096

[24] Jais A Einwallner E Sharif O Gossens K Lu TT-H Soyal SM. Heme oxygenase-1 drives metaflammation and insulin resistance in mouse and man. Cell. (2014) 158:25–40. 10.1016/j.cell.2014.04.043 24995976 PMC5749244

[25] Hu C Li T Xu Y Zhang X Li F Bai J. Cellmarker 2.0: an updated database of manually curated cell markers in human/mouse and web tools based on scRNA-Seq data. Nucleic Acids Res. (2023) 51:D870–6. 10.1093/nar/gkac947 36300619 PMC9825416

[26] Franzén O Gan LM Björkegren JLM. PanglaoDB: a web server for exploration of mouse and human single-cell RNA sequencing data. Database (Oxford). (2019) 2019:baz046. 10.1093/database/baz046 30951143 PMC6450036

[27] Jin S Guerrero-Juarez CF Zhang L Chang I Ramos R Kuan CH. Inference and analysis of cell-cell communication using CellChat. Nat Commun. (2021) 12:1088. 10.1038/s41467-021-21246-9 33597522 PMC7889871

[28] Hao Y Hao S Andersen-Nissen E Mauck WM 3rd Zheng S Butler A. Integrated analysis of multimodal single-cell data. Cell. (2021) 184:3573–3587.e29. 10.1016/j.cell.2021.04.048 34062119 PMC8238499

[29] Chen B Khodadoust MS Liu CL Newman AM Alizadeh AA. Profiling tumor infiltrating immune cells with CIBERSORT. Methods Mol Biol. (2018) 1711:243–59. 10.1007/978-1-4939-7493-1_12 29344893 PMC5895181

[30] Wagner AH Coffman AC Ainscough BJ Spies NC Skidmore ZL Campbell KM. DGIdb 2.0: mining clinically relevant drug-gene interactions. Nucleic Acids Res. (2016) 44:D1036–44. 10.1093/nar/gkv1165 26531824 PMC4702839

[31] Jia C Zhang J Chen H Zhuge Y Chen H Qian F. Endothelial cell pyroptosis plays an important role in kawasaki disease via HMGB1/RAGE/cathespin B signaling pathway and NLRP3 inflammasome activation. Cell Death Dis. (2019) 10:778. 10.1038/s41419-019-2021-3 31611559 PMC6791856

[32] Tada R Nagi-Miura N Adachi Y Ohno N. The influence of culture conditions on vasculitis and anaphylactoid shock induced by fungal pathogen Candida albicans cell wall extract in mice. Microb Pathog. (2008) 44:379–88. 10.1016/j.micpath.2007.10.013 18065191

[33] Lee Y Schulte DJ Shimada K Chen S Crother TR Chiba N. Interleukin-1β is crucial for the induction of coronary artery inflammation in a mouse model of kawasaki disease. Circulation. (2012) 125:1542–50. 10.1161/CIRCULATIONAHA.111.072769 22361326 PMC3337219

[34] Ruelas Castillo J Neupane P Karanika S Krug S Quijada D Garcia A. The heme oxygenase-1 metalloporphyrin inhibitor stannsoporfin enhances the bactericidal activity of a novel regimen for multidrug-resistant tuberculosis in a murine model. Antimicrob Agents Chemother. (2024) 68:e0104323. 10.1128/aac.01043-23 38132181 PMC10848751

[35] Szade A Szade K Mahdi M Józkowicz A. The role of heme oxygenase-1 in hematopoietic system and its microenvironment. Cell Mol Life Sci. (2021) 78:4639–51. 10.1007/s00018-021-03803-z 33787980 PMC8195762

[36] Lee WH Kipp ZA Pauss SN Martinez GJ Bates EA Badmus OO. Heme oxygenase, biliverdin reductase, and bilirubin pathways regulate oxidative stress and insulin resistance: a focus on diabetes and therapeutics. Clin Sci (Lond). (2025) 139:171–98. 10.1042/CS20242825 39873298 PMC12204025

[37] Ni M Zhou J Zhu Z Xu Q Yin Z Wang Y. Shikonin and cisplatin synergistically overcome cisplatin resistance of ovarian cancer by inducing ferroptosis via upregulation of HMOX1 to promote fe(2+) accumulation. Phytomedicine. (2023) 112:154701. 10.1016/j.phymed.2023.154701 36773431

[38] Jiwa H Xie Z Qu X Xu J Huang Y Huang X. Casticin induces ferroptosis in human osteosarcoma cells through fe(2+) overload and ROS production mediated by HMOX1 and LC3-NCOA4. Biochem Pharmacol. (2024) 226:116346. 10.1016/j.bcp.2024.116346 38852641

[39] Blaser H Dostert C Mak TW Brenner D. TNF And ROS crosstalk in inflammation. Trends Cell Biol. (2016) 26:249–61. 10.1016/j.tcb.2015.12.002 26791157

[40] Tian B Nowak DE Brasier AR. A TNF-induced gene expression program under oscillatory NF-kappaB control. BMC Genomics. (2005) 6:137. 10.1186/1471-2164-6-137 16191192 PMC1262712

