# Supplementary material for: Heme oxygenase-1 activation in mononuclear phagocytes in acute Kawasaki disease
Source: Front Pediatr. 2026 Jun 3;14:1848785. doi: 10.3389/fped.2026.1848785 (PMC13272139; doi:10.3389/fped.2026.1848785)
Supplement: Supplementary file 1 [file Datasheet1.pdf]

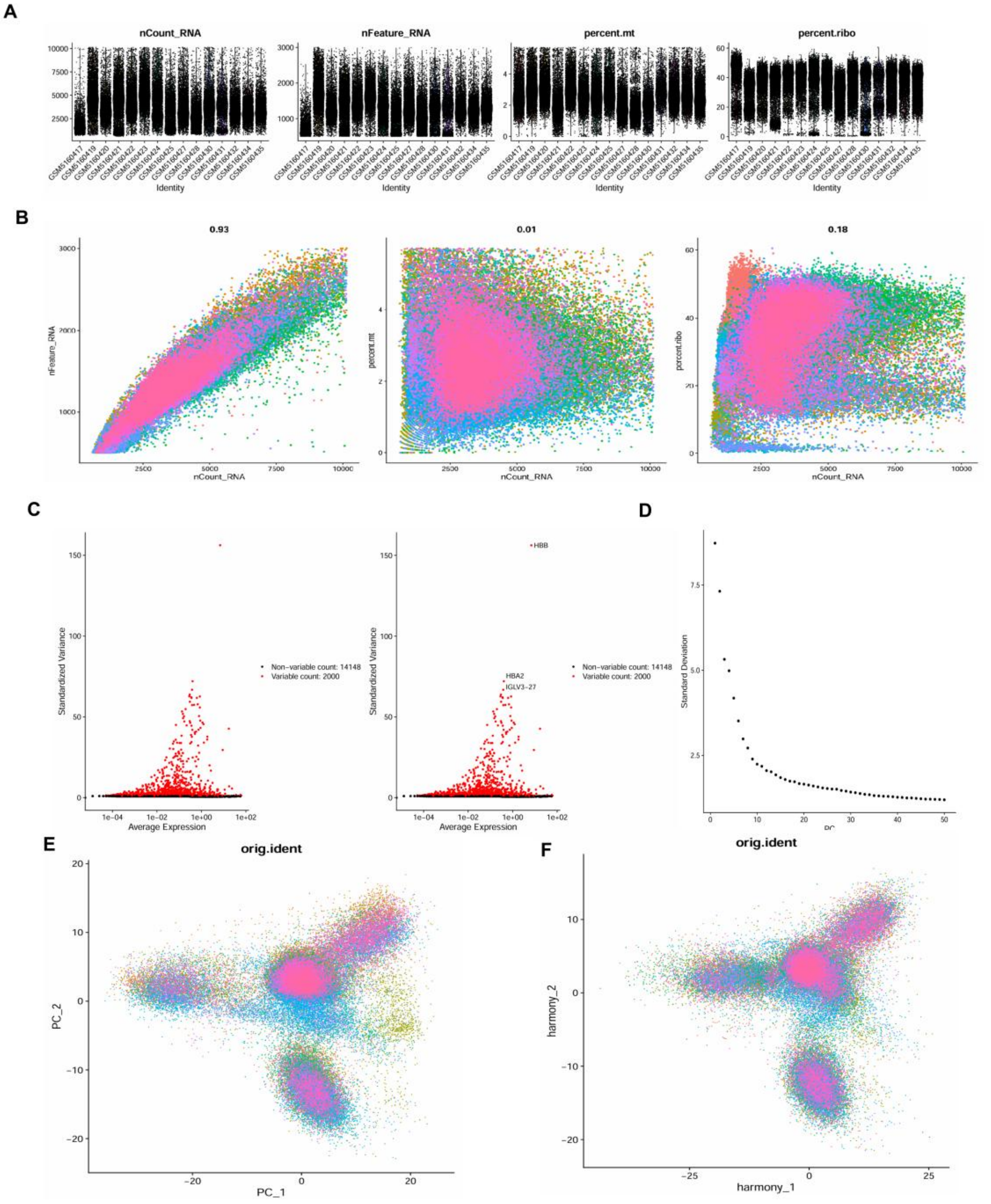

FIGURE S1

Single-Cell Preprocessing (A) Quality control of single cells, illustrating the number of cells, gene count, and sequencing depth for each sample. (B) The left panel depicts the relationship between sequencing depth and mitochondrial content, the middle panel illustrates the correlation between mitochondrial content and nCount_RNA, and the right panel shows the association between sequencing depth and gene quantity. The scatter plot indicates the correlation between mitochondrial content (y-axis) and nCount_RNA (x-axis), with each point representing an individual cell, thereby demonstrating the distribution of RNA counts relative to mitochondrial gene expression levels. (C) We identified genes exhibiting significant intercellular.

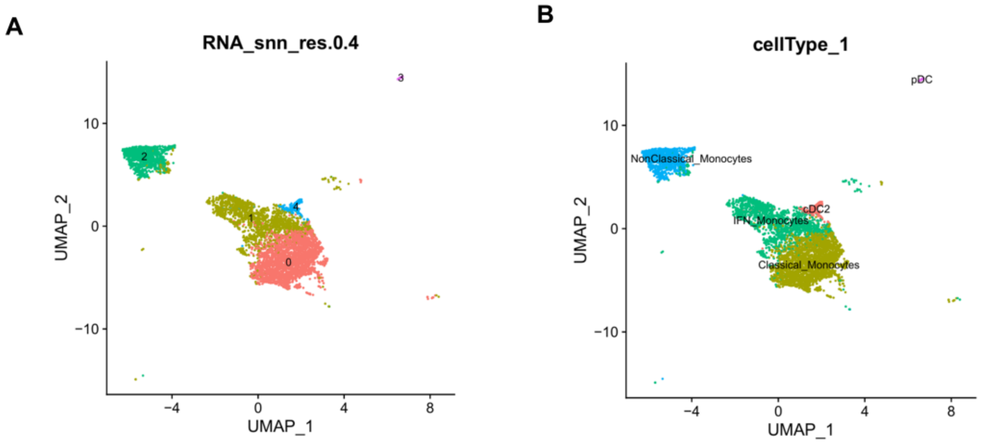

FIGURE S2

Sub-clustering analysis of mononuclear phagocytes. (A)UMAP visualization of sub-clusters within the mononuclear phagocyte compartment extracted from the global PBMC single-cell atlas. (B)Annotation of mononuclear phagocyte subpopulations based on canonical marker gene expression.

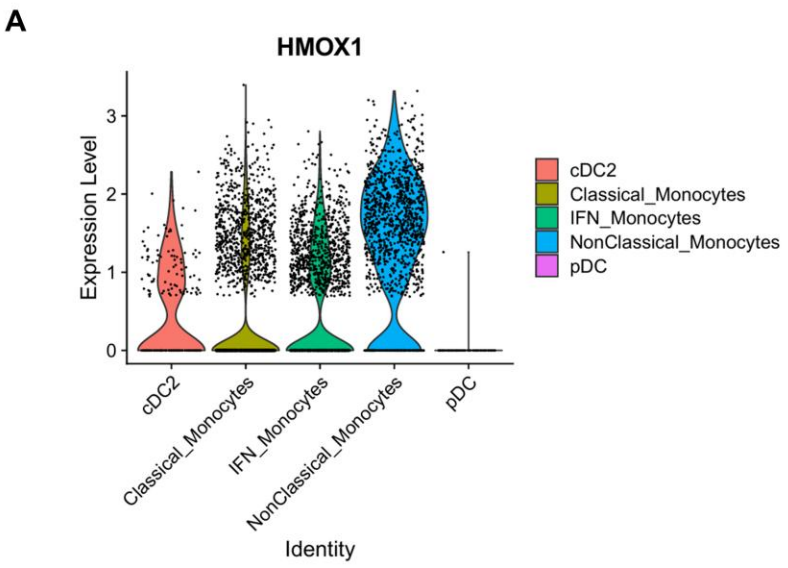

FIGURE S3

Expression of HMOX1 in Mononuclear Phagocyte Subsets.(A)Violin plot showing HMOX1 expression across mononuclear phagocyte subpopulations.

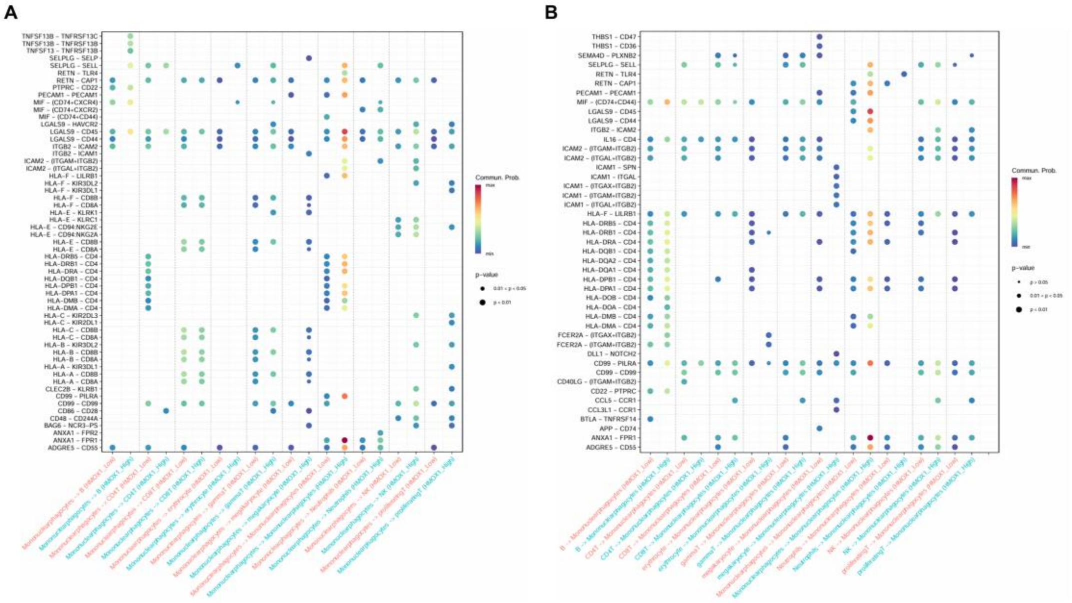

FIGURE S4

CellChat bubble plot analysis of ligand–receptor interactions involving mononuclear phagocytes in the HMOX1 high-expression and low-expression groups. (A) Representative inferred ligand–receptor interactions when mononuclear phagocytes act as signal-sending cells. (B) Representative inferred ligand–receptor interactions when mononuclear phagocytes act as signal-receiving cells.

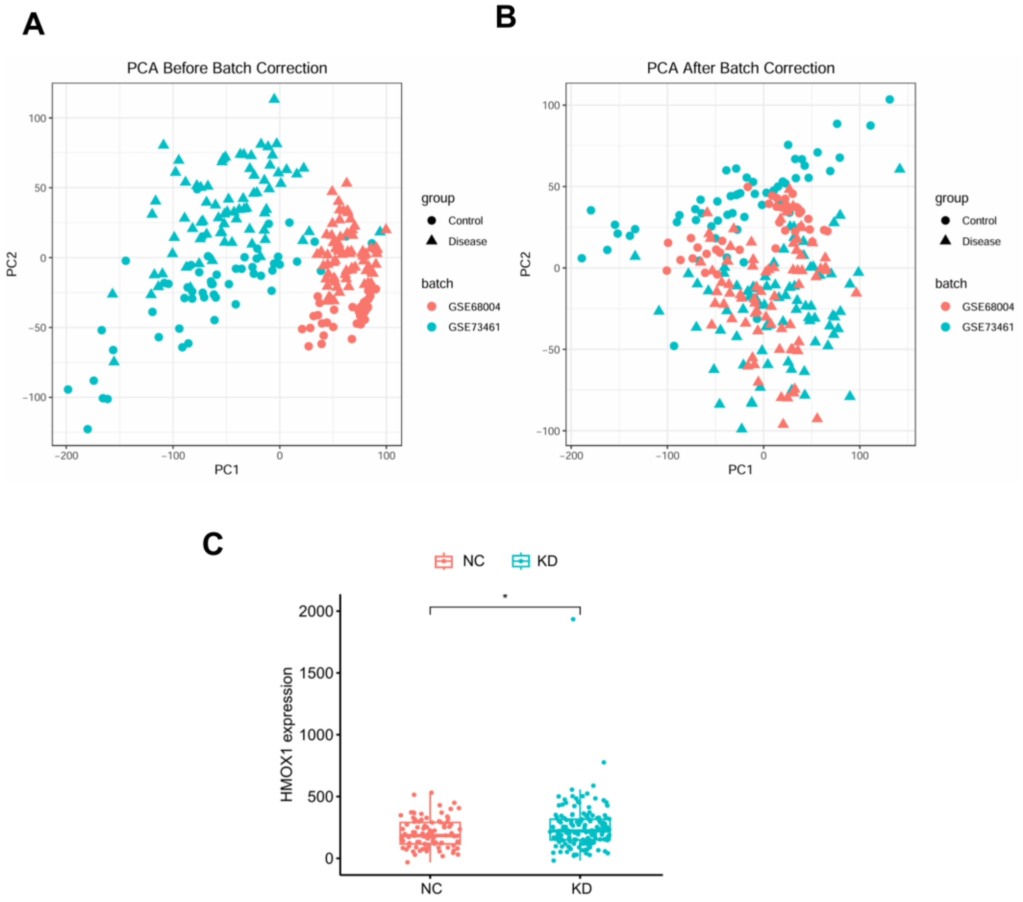

FIGURE S5

Quality Control Assessment of Integrated Kawasaki Disease and Healthy Control Data and Differential Expression Analysis of HMOX1.(A-B) Principal component analysis (PCA) plots that show how batch correction affects datasets from the GEO database. The left panel shows how the samples were spread out before correction. It shows a clear distinction caused by technical batch effects. The right panel shows how the integrated data is spread out after the ComBat algorithm was used. The smaller separation means that the batch effects were effectively removed while the biological signals were kept. (C) Boxplot comparing HMOX1 mRNA expression levels between the normal control group and the Kawasaki illness group. The Y-axis denotes normalized expression values. Statistical analysis indicated a substantial increase in HMOX1 expression in the illness group relative to the control group ($p = 0.021$). The middle line in the box denotes the median, whereas the scatter points signify individual samples.

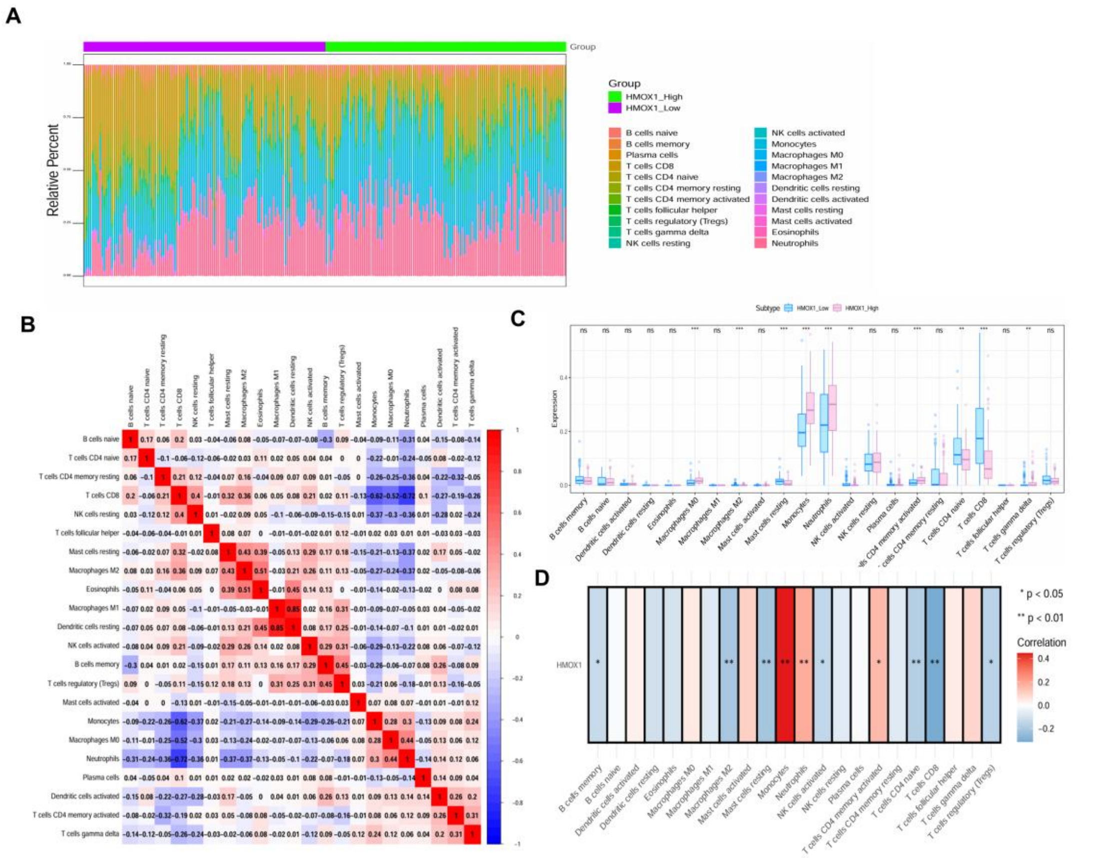

FIGURE S6

Correlation between HMOX1 Expression and Immune Cells. (A) A stacked bar chart illustrates the relative abundance of 22 immune cell subsets in each sample, as determined by the CIBERSORT algorithm, with samples arranged according to HMOX1 high- and low-expression groups. (B) The correlation heatmap illustrates the interaction network among these 22 immune cell types, with blue indicating negative correlations and red indicating positive correlations. (C) Box plots illustrate the levels of immune cell infiltration between the HMOX1 high-expression and low-expression groups, represented in pink and blue, respectively. (D) A bar chart depicts the relationship between HMOX1 expression and the quantity of invading immune cells; the length of each bar represents the correlation coefficient (r), with red indicating positive correlations (e.g., monocytes, neutrophils) and blue indicating negative correlations (e.g., M2 macrophages, quiescent mast cells). Asterisks denote statistical significance (* $p < 0.05$, ** $p < 0.01$).

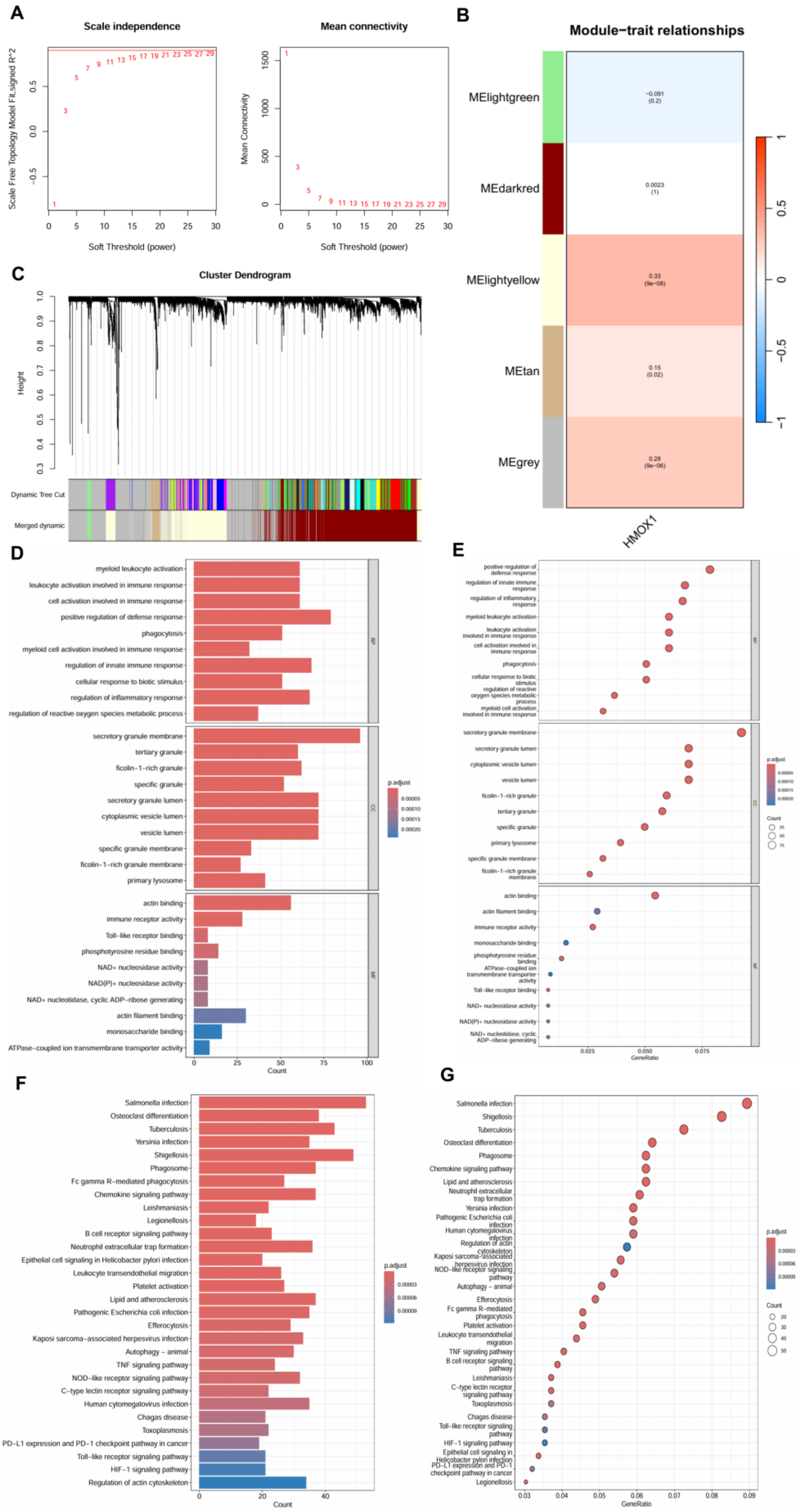
A
Scale independence
Scale Free Topology Model Fit,signed R^2
Soft Threshold (power)
Mean connectivity
Mean Connectivity
Soft Threshold (power)
B
Module−trait relationships
MElightgreen
MEdarkred
MElightyellow
MEtan
MEgrey
−0.091 (0.2)
0.0023 (1)
0.33 (9e−08)
0.15 (0.02)
0.28 (9e−06)
HMOX1
C
Cluster Dendrogram
Height
Dynamic Tree Cut
Merged dynamic
D
myeloid leukocyte activation
leukocyte activation involved in immune response
cell activation involved in immune response
positive regulation of defense response
phagocytosis
myeloid cell activation involved in immune response
regulation of innate immune response
cellular response to biotic stimulus
regulation of inflammatory response
regulation of reactive oxygen species metabolic process
secretory granule membrane
tertiary granule
ficolin−1−rich granule
specific granule
secretory granule lumen
cytoplasmic vesicle lumen
vesicle lumen
specific granule membrane
ficolin−1−rich granule membrane
primary lysosome
actin binding
immune receptor activity
Toll−like receptor binding
phosphotyrosine residue binding
NAD+ nucleosidase activity
NAD(P)+ nucleosidase activity
NAD+ nucleotidase, cyclic ADP−ribose generating
actin filament binding
monosaccharide binding
ATPase−coupled ion transmembrane transporter activity
Count
p.adjust
E
GeneRatio
p.adjust
Count
F
Salmonella infection
Osteoclast differentiation
Tuberculosis
Yersinia infection
Shigellosis
Phagosome
Fc gamma R−mediated phagocytosis
Chemokine signaling pathway
Leishmaniasis
Legionellosis
B cell receptor signaling pathway
Neutrophil extracellular trap formation
Epithelial cell signaling in Helicobacter pylori infection
Leukocyte transendothelial migration
Platelet activation
Lipid and atherosclerosis
Pathogenic Escherichia coli infection
Efferocytosis
Kaposi sarcoma−associated herpesvirus infection
Autophagy − animal
TNF signaling pathway
NOD−like receptor signaling pathway
C−type lectin receptor signaling pathway
Human cytomegalovirus infection
Chagas disease
Toxoplasmosis
PD−L1 expression and PD−1 checkpoint pathway in cancer
Toll−like receptor signaling pathway
HIF−1 signaling pathway
Regulation of actin cytoskeleton
Count
p.adjust
G
GeneRatio
p.adjust
Count

FIGURE S7

Identification of HMOX1-associated co-expression modules via WGCNA and their potential immune regulatory mechanisms. (A) Determination of the soft-thresholding power (β). The left panel plots the scale-free topology fit index across a range of soft-thresholding powers, with the selected threshold (β = 21) for achieving a scale-free network marked by a red line. The right panel displays the corresponding mean connectivity for each power. (B) Clustering dendrogram of all genes based on topological overlap dissimilarity. Distinct colors denote the different modules identified. (C) Heatmap of correlations between module eigengenes and HMOX1 expression. Negative correlations are shown in blue, and positive correlations in red. (D, E) GO enrichment analysis is presented as a bar plot and a bubble plot. Both the bar length and bubble size correspond to the number of enriched genes. A color gradient represents the statistical significance based on the adjusted p-value. (F, G) KEGG enrichment analysis. As in (D, E), the bar length and bubble size indicate the Count, while a color gradient reflects the statistical significance of the enrichment (adjusted p-value).

Supplementary Table 1: Clinical characteristics of children with KD and healthy controls

Characteristic	Control group (n=35)	KD group (n=35)	*p* value
Sex (male/female)	21/14	19/16	0.61
Age (years)	3.24 ± 2.10	3.35 ± 2.05	0.82
Height (cm)	96.50 ± 14.20	97.10 ± 13.80	0.86
Weight (kg)	15.80 ± 4.50	16.10 ± 4.20	0.77
White blood cell count(^10^9/L)	5.67 ± 1.36	21.24 ± 4.31	<0.001
C-reactive protein (mg/L)	2.52 ± 1.02	47.75 ± 7.64	<0.001
Days of fever at the time of blood sampling	—	6.4 ± 1.1	—

Supplemental Table 2: Primer sequences for RT-qPCR

Gene name	Forward primer (5′–3′)	Reverse primer (5′–3′)
GAPDH	CGGAGTCAACGGATTTGGTCGTAT	AGCCTTCTCCATGGTGGTGAAGA
HMOX1	CCAGGCAGAGAATGCTGAGTTC	AAGACTGGGCTCTCCTTGTTGC
